# The Effectiveness of Duloxetine for Knee Osteoarthritis: An Overview of Systematic Reviews

**DOI:** 10.3389/fphys.2022.906597

**Published:** 2022-06-07

**Authors:** Qinxin Zhou, Jixin Chen, Weijie Yu, Kun Yang, Tianci Guo, Puyu Niu, Yuntian Ye, Aifeng Liu

**Affiliations:** ^1^ Department of Orthopaedic Surgery, First Teaching Hospital of Tianjin University of Traditional Chinese Medicine, Tianjin, China; ^2^ National Clinical Research Center for Chinese Medicine Acupuncture and Moxibustion, Tianjin, China

**Keywords:** duloxetine, knee osteoarthritis, depression, overview, systematic review, methodological quality

## Abstract

**Background:** Knee osteoarthritis (KOA) has become a public health problem. Several systematic reviews (SRs) have reported that duloxetine may be an effective treatment for improving pain and depressive symptoms in patients with KOA.

**Aim:** To evaluate the available results and provide scientific evidence for the efficacy and safety of duloxetine for KOA.

**Methods:** A comprehensive search strategy was conducted across eight databases from inception to 31 December 2021. Two researchers independently selected eligible studies, collected data and evaluated those included SRs’ quality. For assessing methodological quality, the Assessing the Methodological Quality of Systematic Reviews 2 (AMSTAR 2) was employed. Risk of Bias in Systematic Reviews (ROBIS) was used to assess the risk of bias. Preferred Reporting Items for Systematic Review and Meta-analyses (PRISMA) was utilized for assessing reporting quality. In addition, the Grading of Recommendations Assessment, Development, and Evaluation (GRADE) was used to determine primary outcome indicators’ evidence quality.

**Results:** Totally 6 SRs were contained in this overview. After assessment based on AMSTAR 2, ROBIS, and PRISMA, unsatisfactory results in terms of methodological quality, risk of bias as well as reporting quality, were obtained. Limitations included a search of grey literature, the reasons for selecting the study type, an excluded study list and the specific reasons, reporting bias assessment, and reporting of potential sources of conflict of interest. According to the GRADE results, the evidence quality was high in 0, moderate in 5, low in 19, and very low in 36. Limitations were the most commonly downgraded factor, followed by publication bias and inconsistency.

**Conclusion:** Duloxetine may be an effective treatment for improving pain and depressive symptoms in KOA patients with acceptable adverse events. However, due to the low quality of the available evidence, the original study design and the quality of evidence from SRs should be further improved, so as to provide strong scientific evidence for definitive conclusions.

**Systematic Review Registration:** PROSPERO; (http://www.crd.york.ac.uk/PROSPERO/), identifier (CRD42021289823).

## 1 Introduction

Knee osteoarthritis (KOA) refers to one of the most frequent joint diseases, characterized by progressive cartilage loss, subchondral bone remodeling and synovial inflammation, causing symptoms such as chronic pain, joint stiffness as well as physical and psychological disturbances (Pigeolet et al., 2021; Sharma, 2021). Over the past 20 years, around 250 million people across the world have been diagnosed with KOA, and the global prevalence has increased significantly (GBD 2015 Disease and Injury Incidence and Prevalence Collaborators, 2015). In addition to chronic pain and disability, nearly 21% of adults undergoing KOA suffer from depression, and the relative risk of depression in people with KOA compared to those without KOA is 1.17 (Kessler et al., 2003; Stubbs et al., 2016). In patients suffering from KOA, including depression, the depressive or anxious mood is associated with higher levels of pain (Axford et al., 2010). They were reported to have higher healthcare utilization costs and more frequent use of pain medication due to low awareness of depression (Gleicher et al., 2011). In addition, this group of patients were also less probably to fully comply with the recommended treatment regimens than KOA patients with undiagnosed depression, thereby increasing the burden of illness and the difficulty of management (Sale et al., 2008). Current guidelines have evaluated over 50 treatments for osteoarthritis of the knee (Bannuru et al., 2019; Kolasinski et al., 2020). Oral medications contain acetaminophen, NSAIDs, and strong and weak opioids. The guidelines recommend paracetamol as a first-line drug and NSAIDs and opioids as second and third-line drugs. However, there are still reservations in association with the long-term safety and efficacy of NSAIDs and opioids (Bruyère et al., 2019; Arden et al., 2021).

Duloxetine refers to a 5-hydroxytryptamine and noradrenaline reuptake inhibitor that treats pain using the downstream pain modulation system (Ferreira et al., 2021). Guidelines for osteoarthritis, such as the Osteoarthritis Research Society International (OARSI) and the American College of Rheumatology, recommend the application of duloxetine for pain management (Bannuru et al., 2019; Kolasinski et al., 2020). Chronic pains associated with osteoarthritis involve dysfunction of central pain pathways in line with researches about the pathophysiology of KOA pain (Malfait and Schnitzer, 2013; Miller et al., 2014). Studies have demonstrated that imbalances in the 5-hydroxytryptamine and norepinephrine systems within the central pain pathway exert a vital function in the onset of pain sensitization (Miller et al., 2017; Bannuru et al., 2019). Therefore, duloxetine may be a better treatment. In animal models of central sensitization to KOA, duloxetine is effective in relieving persistent pain (Havelin et al., 2016). Duloxetine is currently being clinically applied for the treatment of KOA and has exhibited good symptom relief (Wang et al., 2017; Uchio et al., 2018; Koh et al., 2019). The number of clinical studies and SRs reporting the efficacy of duloxetine for the treatment of KOA is increasing. As a top element of the evidence pyramid, SRs are often considered to aid in identifying, evaluating, and synthesizing study-based evidence in order to assist with clinical decision-making (Siddaway et al., 2019). Nevertheless, the conclusions of these SRs are controversial due to the irregular reporting, methodological flaws, and low-quality evidence. Meanwhile, their clinical guidance needs to be further validated. Only high-quality evidence-based medical evidence is reliable, while low-quality evidence can instead generate mislead clinicians. An overview of SRs is a comprehensive approach to evaluating studies across multiple SRs and synthesizing evidence (Thomson et al., 2010; Smith et al., 2011; Baker et al., 2014; Huang et al., 2021).

To our knowledge, this overview of SRs is the first attempt with the purpose of assessing the efficacy and safety of duloxetine SRs objectively and comprehensively in enhancing pain and depressive symptoms in patients undergoing KOA. We aim to provide a scientific basis for clinicians, decision-makers and patients with KOA as well as a basis for guidance for future SR producers.

## 2 Methods

### 2.1 Protocols and Registration

A predetermined written protocol of the current overview was registered in the PROSPERO database with the registration number: CRD42021289823.

### 2.2 Search Strategy

Two independent researchers conducted electronic literature searches in four international electronic databases (PubMed, EMBASE, Cochrane Library, and Web of Science) and four Chinese electronic databases (Chinese National Knowledge Infrastructure, Chinese Biological Medicine, WanFang and Chongqing VIP database) from the inception to 31 December 2021. In addition, the research registry, relevant grey literature and consultation with experts in the relevant fields were further searched manually. No language restriction was applied. This study utilized the following search terms, including (“osteoarthritis of the knee” OR “knee osteoarthritis” OR “koa” OR “gonarthritis” OR “knee pain”) AND (“duloxetine” OR “duloxetine hydrochloride’’ OR “Cymbalta”) AND (“systematic review” OR “systematic evaluation” OR “meta-analyses” OR “meta-analysis”). Apart from that, the search strategy was illustrated by PubMed (Table 1).

**TABLE 1:** Search strategy for PubMed database.

Query	Search item
# 1	Osteoarthritis, Knee (Mesh)
# 2	Osteoarthritis, Knee (Title/Abstract)
# 3	Knee osteoarthritis (Title/Abstract)
# 4	Knee osteoarthritides (Title/Abstract)
# 5	Knee pain (Title/Abstract)
# 6	Knee joint osteoarthritis (Title/Abstract)
# 7	Knee arthritis (Title/Abstract)
# 8	Osteoarthritis of knee (Title/Abstract)
# 9	KOA (Title/Abstract)
# 10	Gonarthrosis (Title/Abstract)
# 11	Osteoarthrosis (Title/Abstract)
# 12	# 1 OR # 2–11
# 13	Duloxetine hydrochloride (MeSH)
# 14	Duloxetine (Title/Abstract)
# 15	Cymbalta (Title/Abstract)
# 16	# 13 OR # 14–15
# 17	Meta-analysis (Publication Type)
# 18	Meta-analysis (MeSH)
# 19	Systematic evaluation (Title/Abstract)
# 20	Systematic review (Title/Abstract)
# 21	Meta analysis (Title/Abstract)
# 22	Meta analyses (Title/Abstract)
# 23	# 17 OR # 18–22
# 24	# 12 AND # 16 AND # 23

### 2.3 Inclusion Criteria

This study included SRs matched with the following criteria: 1) Study design: SRs of RCTs reporting the effects of duloxetine on KOA. To be eligible for this overview, several restrictions were applied on SRs. Besides, a comprehensive search strategy was conducted using 5 or more databases. RCTs in the included SRs should conduct at least 2-weeks duloxetine interventions with >10 patients in each group. SRs were reported according to the PRISMA statement guidelines, with quantitative synthesis (meta-analysis) and language restricted to Chinese and English. 2) Study participants met the KOA diagnostic criteria of the American College of Rheumatology, regardless of gender, age, race, nationality, or disease duration. 3) Study intervention: the treatment group adopted duloxetine as the main drug, while the control group used standard drug treatment without duloxetine, placebo, or no treatment. 4) Study outcome measures included Brief Pain Inventory-Severity (BPI-S), Patient Global Improvement-Inventory (PGI-I), Western Ontario and McMaster Universities score total score (WOMAC), WOMAC pain score, WOMAC physical function score, WOMAC stiffness score, 30% reduction and 50% reduction.

### 2.4 Exclusion Criteria

Repeated publications; non-SRs; the control group using duloxetine as the treatment; conference abstracts.

### 2.5 Literature Screening and Data Extraction

In accordance with the search strategy, two researchers imported the retrieved titles into Endnote software. After the removal of duplicates, titles and abstracts of articles detected in the search are screened independently by two members and categorized as included, unclear or exclude. The full reports of all articles that categorized as included or unclear are examined regarding the compliance of reviews with eligibility criteria. If there was any dispute, they discussed and agreed, or the third member decided whether to include it or not. Based on the data extracted from SRs by two independent members, the following could be summarized including first author’s initials, publication year, number of included RCTs, sample size, interventions in the treatment and control groups, a tool for assessing quality, adverse events, outcomes as well as main conclusions of the included SRs.

### 2.6 Review Quality Assessment

The quality evaluation of this overview mainly followed the Cochrane Handbook and the methods of relevant high systematic evaluation re-evaluation studies. The quality evaluation mainly contained four aspects of evaluation, respectively, methodological quality, report quality, evidence quality and risk of bias and was performed by two investigators independently. If differences were encountered, the consensus was achieved through negotiation, and a third party ruled if necessary.

#### 2.6.1 Methodological Quality Evaluation

Evaluation of the methodology quality of the included SRs was done based on the AMSTAR 2 tool, which is a comprehensive critical appraisal instrument to evaluate SRs of randomized trials (Shea et al., 2017). The contained studies were rated as high quality according to the criteria of “no or only 1 non-critical entry non-conformity,” “more than 1 non-critical entry non-conformity” and “moderate quality”. The included studies were rated as high, medium, low, and very low quality in line with the criteria of “no or one non-critical entry non-conformity,” “more than one non-critical entry non-conformity,” “one non-critical entry non-conformity,” “one non-critical entry non-conformity,” “one non-critical entry non-conformity,” “low quality,” and “very low quality”. In addition, totally16 entries were evaluated, including seven key entries, namely, entries 2, 4, 7, 9, 11, 13, and 15.

#### 2.6.2 Risk of Bias Evaluation

The risk of bias evaluation of the included SRs was performed using 24 entries in the ROBIS tool, which is the first rigorously developed tool designed particularly in order to evaluate the risk of bias in SRs (Whiting et al., 2016). The instrument is finished in 3 phases, which can assist in judging the risk of bias during the process of review, results as well as conclusions. Each entry was described by the authors, and responses to all questions were indicated by “yes,” “probably yes,” “could be,” “no,” and “no information”. Finally, the risk of bias in the field was judged as “low,” “high,” or “uncertain”. The risk of bias in this area is “low” if all the landmark questions are answered as “yes” or “probably yes”. If any of the landmark questions are answered by “maybe” or “could be,” the risk of bias in this area is “low”. If the answers to any of the landmark questions were “may or may not” or “no”, the risk of bias was “high”. If the offered information was inadequate to make a judgment, the risk of bias was “uncertain”. In addition, the risk of bias was “uncertain” if the information provided was not sufficient.

#### 2.6.3 Report Quality Evaluation

The PRISMA statement is a reporting guidance that reflects advances in methods with the purpose of identifying, selecting, appraising, and synthesizing studies and can be adopted for evaluating the reporting quality in the contained SRs (28). The PRISMA statement list is consisted of 27 entries, including seven perspectives of SRs, respectively, title, abstract, introduction, methods, results, discussion, and funding (Page et al., 2021). The answer options for each item contain “yes,” “no,” and “partial yes”. The completion of each project is denoted as a ratio. In addition, the PRISMA statement claims that reports with completeness of less than 50% of each entry are considered to have a deficiency in the reported information.

#### 2.6.4 GRADE Evidence Quality Evaluation

The quality of evidence for each outcome indicator involved in the SRs was assessed by the GRADE tool, with limitations, inconsistency, non-directness, imprecision as well as publication bias as downgrading factors (Guyatt et al., 2008). Apart from that, the quality of evidence was regarded to have high quality with no downgrading, moderate quality with 1 downgrade, low quality with 2 downgrades as well as very low quality with 3 downgrades and above. The current work carried out a descriptive analysis of extracting findings from the contained researches concerning the effectiveness and safety of duloxetine in treating KOA.

### 2.7 Data Synthesis and Presentation

A narrative synthesis was applied in the current overview. In addition, the features and results of each SR as well as the findings of AMSTAR 2, ROBIS, GRADE, and PRISMA were shown in tables and figures.

## 3 Results

### 3.1 Literature Search

**FIGURE 1:**
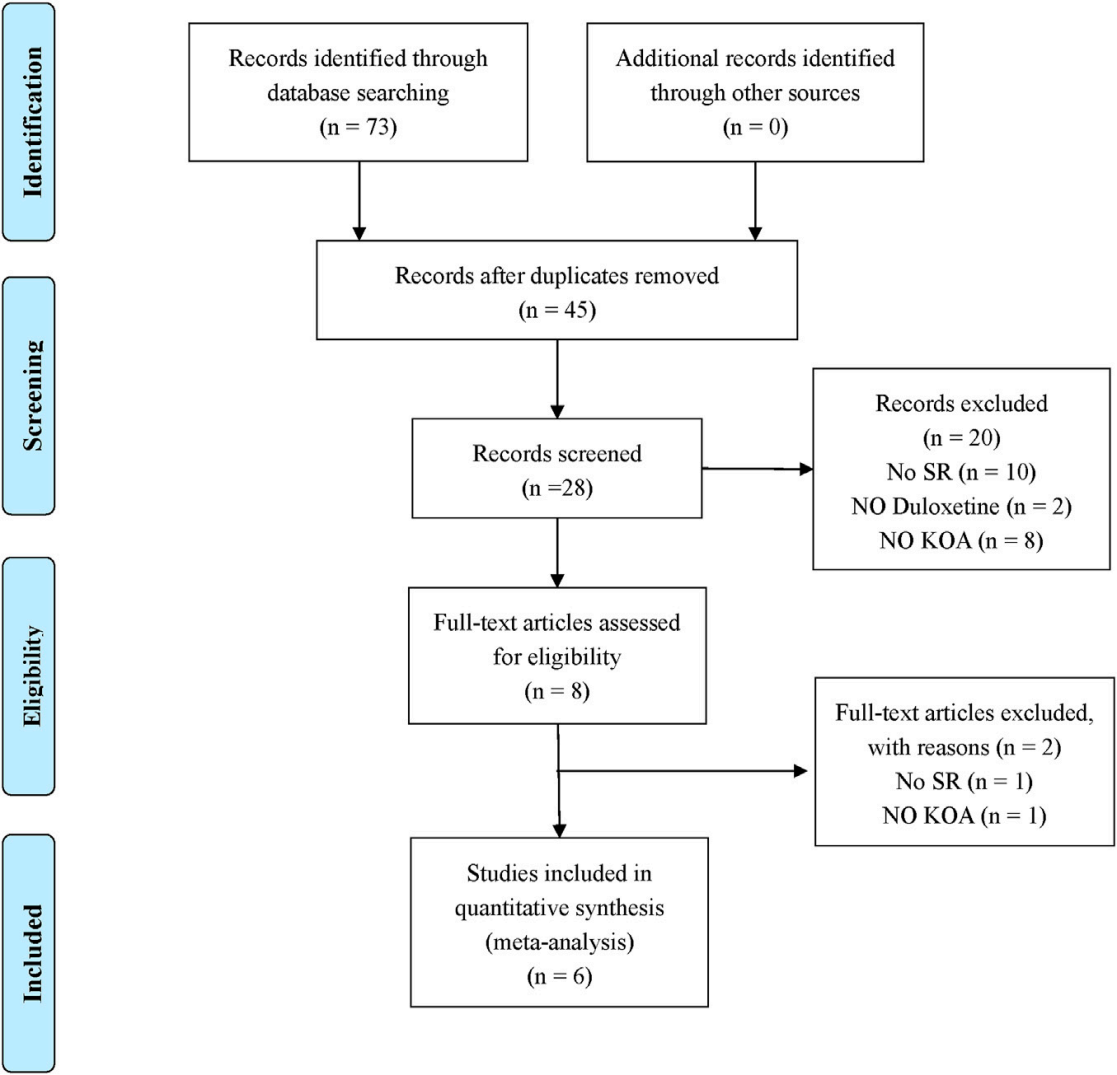
Literature selection procedure.

According to the search strategy, 73 original titles were initially examined, including 12 from Chinese National Knowledge Infrastructure, 3 from WanFang database, 1 for Chongqing VIP database, 0 from Chinese Biological Medicine, 16 from PubMed, 17 from EMBASE, 0 from The Cochrane Library, and 24 from Web of Science. In addition, the title list was imported into Endnote software. After the duplicates were screened out, eight articles were left. After referring to the full text, six papers were finally included (Wang et al., 2015; Chen et al., 2019; Gao et al., 2019; Osani and Bannuru, 2019; Qu et al., 2020; Chen et al., 2021). The literature screening process is detailed in Figure 1.

### 3.2 Basic Features of Included Literature

Totally 6 SRs were included in this study, all of which have been published between 2015–2021, with 3 published in 2019. The number of RCTs in the SRs ranged from 3 to 6. Among them, five were published in English (Wang et al., 2015; Chen et al., 2019; Gao et al., 2019; Osani and Bannuru, 2019; Chen et al., 2021) and one in Chinese (Qu et al., 2020). Among the risk of bias assessment tools for RCTs, one paper chose the Jadad score (Wang et al., 2015) and five papers selected the Cochrane Handbook recommended risk of bias assessment tools (Chen et al., 2019; Gao et al., 2019; Osani and Bannuru, 2019; Qu et al., 2020; Chen et al., 2021). In addition, six papers decided on duloxetine 60/120 mg, with Qd as the intervention group and placebo as the control group, finding that duloxetine improved pain and function in KOA patients while attention is required to be paid to the occurrence of adverse events. Table 2 presents the basic characteristics of the included studies.

**TABLE 2:** Characteristics of the included SRs.

Author (year)	Country	Number of RCT (Total population)	Intervention		Outcome measures	Quality assessment tool	Overall conclusion
			Treatment group	Control group			
Wang (2015)	China	3 (*n* = 1001)	Duloxetine 60/120 mg, Qd	Placebo	BPI-S, 30% pain reduction rate, 50% pain reduction rate, PGI-I, WOMAC physical function score, AEs, TFAEs, SAEs, TDR	Jadad score	This analysis suggests duloxetine [60/120 mg, quaque die (Qd)], compared with placebo control, resulted in a greater reduction in pain, improved function and patient-rated impression of improvement, and acceptable adverse effects for the treatment of OAK pain after approximately 10–13 weeks of treatment.
Chen (2019)	China	6 (*n* = 2059)	Duloxetine 60/120 mg, Qd	Placebo	BPI-S, weekly 24-h average pain score, 30% pain reduction rate, 50% pain reduction rate, WOMAC stiffness score, WOMAC physical function score, TFAEs, SAEs, TDR	Cochrane risk of bias tool	Duloxetine is effective in the management of chronic pain and loss of physical function in knee OA with acceptable adverse events despite having no advantage in treating joint stiffness. Future trials should focus on determining the optimal treatment regimen.
Gao (2019)	China	5 (*n* = 1774)	Duloxetine 60/120 mg, Qd	Placebo	BPI-S, 30% pain reduction rate, 50% pain reduction rate, PGI-I, WOMAC total score, WOMAC pain score, WOMAC stiffness score, WOMAC physical function score, TFAEs, SAEs, TDR	Cochrane risk of bias tool	Duloxetine was an effective and safe choice to improve pain and functional outcome in OA patients. However, further studies are still needed to find out the optimal dosage for OA and examine its long-term efficacy and safety.
Osani (2019)	America	5 (*n* = 1713)	Duloxetine 60/120 mg, Qd	Placebo	WOMAC pain score, WOMAC physical function score, TFAEs, SAEs, TDR, Gastrointestinal adverse event, Quality of life improvement, Improvement of depressive symptoms	Cochrane risk of bias tool	Duloxetine may be an effective treatment option for individuals with knee OA, but use of the drug is associated with a significantly higher risk of adverse events
Qu (2020)	China	6 (*n* = 2059)	Duloxetine 60/120 mg, Qd	Placebo	BPI-S, WOMAC total score, WOMAC pain score, WOMAC stiffness score, WOMAC physical function score, Dry mouth, Drowsiness, Nausea	Cochrane risk of bias tool	Duloxetine can relieve pain and improve knee function in PATIENTS with KOA, but it is necessary to pay attention to the occurrence of adverse reactions
Chen (2021)	China	6 (*n* = 2059)	Duloxetine 60/120 mg, Qd	Placebo	BPI-S, BPI-I, 30% pain reduction rate, 50% pain reduction rate, Pain reduction average rate, PGI-I, CGI-I, WOMAC pain score, WOMAC stiffness score, WOMAC physical function score, TFAEs, SAEs, SF-36 physical functional subscale, SF-36 bodily pain subscale, SF-36 role physical subscale	Cochrane risk of bias tool	Duloxetine may be an effective treatment option for knee OA patients but further rigorously designed and well-controlled randomized trials are warranted.

Abbreviations: AEs, adverse events; BPI-I, Brief Pain Inventory-Interference; BPI-S, Brief Pain Inventory-Severity; CGI-S, Clinical Global Impressions of Severity; PGI-I, Patient’s Global Impression of Improvement; SAEs, Serious adverse events; SF-36, 36-Item Short-Form Health Status Survey; TDR, Treatment discontinuation rate; TEAEs, treatment-emergent adverse events; WOMAC, Western Ontario and McMaster Universities Osteoarthritis Index.

### 3.3 Results of Review Quality Assessment

#### 3.3.1 Methodological Quality

Table 3 showed the findings of methodological quality assessed by AMSTAR 2 tool. All SRs were rated to be the critical low quality. For the critical items, 3 SRs (Wang et al., 2015; Osani and Bannuru, 2019; Qu et al., 2020) reported either predefined protocol (item 2). No SR reported the comprehensive search strategy (item 4), offered the list of excluded studies and provided the reasons for exclusion (item 7) completely. When it came to the evaluation of risk of bias, 5 SRs considered random sequence allocation as well as the selection of the outcome report (item 9). In terms of statistical combination, 6 SRs (100%) integrated the result with suitable methods (item 11) and explained RoB in individual studies while exploring the results (item 13). The last critical item (item 15) was associated with publication bias and 2 SRs (Wang et al., 2015; Osani and Bannuru, 2019) reported it fully. Items 3, 5, 12, and 16 were rated especially low quality. In addition, all SRs had chosen RCT, without accounting for the causes of selection. No SR reported the potential sources of conflicts of interest containing the funding sources for the studies.

**TABLE 3:** Results of the AMSTAR 2 assessments.

Author (year)	AMSTAR 2																	Overall quality
	Q1		Q2	Q3	Q4	Q5	Q6	Q7	Q8	Q9	Q10	Q11	Q12	Q13	Q14	Q15	Q16	
Wang (2015)		Y	N	N	PY	N	Y	N	PY	PY	Y	Y	N	Y	Y	Y	N	Critically low
Chen (2019)		Y	Y	N	PY	N	Y	N	PY	Y	Y	Y	N	Y	Y	N	N	Critically low
Gao (2019)		Y	Y	N	PY	N	Y	N	PY	Y	Y	Y	N	Y	Y	N	N	Critically low
Osani (2019)		Y	N	N	PY	N	Y	N	PY	Y	N	Y	N	Y	Y	Y	N	Critically low
Qu (2020)		Y	N	N	PY	N	Y	N	PY	Y	Y	Y	N	Y	Y	N	N	Critically low
Chen (2021)		Y	Y	N	PY	N	Y	N	PY	Y	Y	Y	N	Y	Y	N	N	Critically low
Number of Y (%)		6 (100)	3 (50)	0 (0)	0 (0)	0 (0)	6 (100)	0 (0)	0 (0)	5 (83.3)	5 (83.3)	6 (100)	0 (0)	6 (100)	6 (100)	2 (33.3)	0 (0)	

Abbreviations: Y, Yes; PY, Partial Yes; N, No.

Q1: Did the research questions and inclusion criteria for the review include the components of PICO?

Q2: Did the report of the review contain an explicit statement that the review methods were established prior to the conduct of the review and did the report justify any significant deviations from the protocol?

Q3: Did the review authors explain their selection of the study designs for inclusion in the review?

Q4: Did the review authors use a comprehensive literature search strategy?

Q5: Did the review authors perform study selection in duplicate?

Q6: Did the review authors perform data extraction in duplicate?

Q7: Did the review authors provide a list of excluded studies and justify the exclusions?

Q8: Did the review authors describe the included studies in adequate detail?

Q9: Did the review authors use a satisfactory technique for assessing the risk of bias (RoB) in individual studies that were included in the review?

Q10: Did the review authors report on the sources of funding for the studies included in the review?

Q11: If meta-analysis was performed did the review authors use appropriate methods for statistical combination of results?

Q12: If meta-analysis was performed, did the review authors assess the potential impact of RoB in individual studies on the results of the meta-analysis or other evidence synthesis?

Q13: Did the review authors account for RoB in individual studies when interpreting/discussing the results of the review?

Q14: Did the review authors provide a satisfactory explanation for, and discussion of, any heterogeneity observed in the results of the review?

Q15: If they performed quantitative synthesis did the review authors carry out an adequate investigation of publication bias (small study bias) and discuss its likely impact on the results of the review?

Q16: Did the review authors report any potential sources of conflict of interest, including any funding they received for conducting the review?

### 3.4 Risk of Bias of Included SRs

**FIGURE 2:**
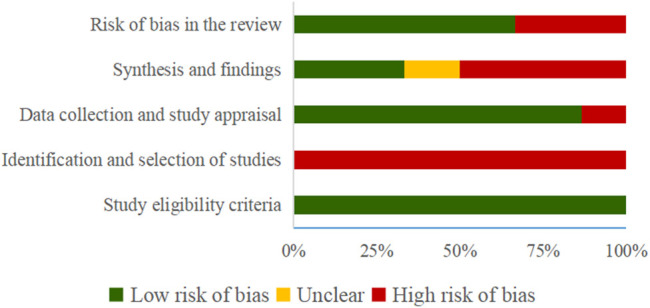
Risk of bias of the included SRs with ROBIS tool.

The results of the risk of bias evaluated by ROBIS tool demonstrated that all SRs were rated as low risk in Domain 1 of Phase 2 (study eligibility criteria). In terms of Domain 2, by evaluating the identification and selection of studies, 6 (100%) SRs were rated to be low-risk. 1 SR (Wang et al., 2015) was rated as high risk in Domain 3 (data collection and study appraisal) with 3 SRs (Chen et al., 2019; Gao et al., 2019; Qu et al., 2020) being rated as high risk in Domain 4 (synthesis and findings). Finally, 2 SRs (Chen et al., 2019; Gao et al., 2019) were rated to be low-risk in Phase 3 (risk of bias in the review). Table 4 and Figure 2 show the detailed results.

**TABLE 4:** Results of the ROBIS tool.

Review	Phase 2				Phase 3
	1.Study eligibility criteria	2.Identification and selection of studies	3.Data collection and study appraisal	4.Synthesis and findings	Risk of bias in the review
Wang (2015)	☺	☹	☹	☺	☺
Chen (2019)	☺	☹	☺	☹	☹
Gao (2019)	☺	☹	☺	☹	☹
Osani (2019)	☺	☹	☺	?	☺
Qu (2020)	☺	☹	☺	☹	☺
Chen (2021)	☺	☹	☺	☺	☺

Abbreviations ☺ = low risk of bias; ☹ = high risk of bias; ? = unclear.

### 3.5 Reporting Quality of Included SRs

The results of reporting quality assessed by PRISMA checklists were shown in Table 5. The reporting of the titles, introductions, and discussions of the SRs included is complete (100%). However, some entries were reported to be deficient (<50%) such as item 2 (abstract), item 6 (Information sources), item 7 (Search strategy), item 10 (Data items), item 14 (Reporting bias assessment), item 15 (Certainty assessment), item 16 (Study election), item 20 (Results of syntheses), and item 27 (Availability of data, code and other materials).

**TABLE 5:** Results of the PRISMA assessments.

Section/topic	Items	Wang (2015)	Chen (2019)	Gao (2019)	Osani (2019)	Qu (2020)	Chen (2021)	Compliance (%)
Title								
	1.Title	Y	Y	Y	Y	Y	Y	100
Abstract								
	2.Abstract	PY	PY	PY	PY	PY	PY	0
Introduction								
	3.Rationale	Y	Y	Y	Y	Y	Y	100
	4.Objectives	Y	Y	Y	Y	Y	Y	100
Methods								
	5.Eligibility criteria	Y	Y	Y	Y	Y	Y	100
	6.Information sources	PY	PY	PY	PY	PY	PY	0
	7.Search strategy	N	N	N	N	N	N	0
	8. Selection process	Y	Y	Y	Y	Y	Y	100
	9.Data collection process	Y	Y	Y	Y	Y	Y	100
	10.Data items	PY	PY	PY	PY	PY	PY	0
	11.Study risk of bias assessment	Y	Y	Y	Y	Y	Y	100
	12.Effect measures	Y	Y	Y	Y	Y	Y	100
	13.Synthesis methods	PY	PY	PY	PY	PY	PY	0
	14.Reporting bias assessment	Y	N	N	Y	N	N	66.7
	15.Certainty assessment	N	N	Y	N	N	N	16.7
Results								
	16.Study election	PY	PY	PY	PY	PY	PY	0
	17.Study characteristics	Y	Y	Y	Y	Y	Y	100
	18.Risk of bias within studies	N	Y	Y	Y	Y	Y	83.3
	19.Results of individual studies	Y	Y	Y	N	Y	Y	83.3
	20.Results of syntheses	PY	PY	PY	Y	PY	PY	16.7%
	21.Reporting biases	N	Y	Y	Y	N	N	50%
	22.Certainty of evidence	Y	Y	Y	Y	Y	Y	100%
Discussion								
	23.Discussion	Y	Y	Y	Y	Y	Y	100%
Other information								
	24.Registration and protocol	N	Y	Y	N	N	Y	50%
	25.Support	Y	Y	Y	N	Y	Y	83.3%
	26.Competing interests	Y	Y	Y	Y	N	Y	83.3%
	27.Availability of data, code and other materials	PY	PY	PY	PY	PY	PY	0%

Abbreviations: Y, yes (a complete report); PY, partially yes (a partially compliant report); N, no (no report).

### 3.6 Evidence Quality Grading

The findings of evidence quality rated by GRADE were presented in Table 6. The included SRs had a total of 60 outcome indicators. 6 SRs were initially graded as high in evidence because they included RCTs, and were rated for five downgrading factors, respectively, limitations (*n* = 60, 100%), publication bias (*n* = 43, 71.7%), inconsistency (*n* = 32, 53.3%), imprecision (*n* = 28, 46.7%), and indirectness (*n* = 0, 0%). The final results revealed that none was high quality, 5 (8.3%) were moderate quality, 19 (31.7%) were low quality, and 36 (60%) were critically low quality.

**TABLE 6:** GRADE quality grading of included SRs.

Author (year)	Outcomes (n)	Limitations	Inconsistency	Indirectness	Imprecision	Publication bias	Quality of evidence
Wang (2015)	WOMAC physical function score (3)	−1^①^	0	0	−1^③^	0	L
	PGI-I scores (3)	−1^①^	−1^②^	0	0	0	L
	30% pain reduction rate (3)	−1^①^	0	0	0	0	M
	50% pain reduction rate (3)	−1^①^	−2^②^	0	−1^③^	0	CL
	BPI-S score (3)	−1^①^	0	0	0	0	M
	Adverse events (3)	−1^①^	0	0	−1^③^	0	L
	Serious adverse events (3)	−1^①^	0	0	−1^③^	0	L
	Treatment emergent adverse events (3)	−1^①^	0	0	−1^③^	0	L
	Treatment discontinuation rate (3)	−1^①^	−1^②^	0	−1^③^	0	CL
Chen (2019)	WOMAC stiffness score (6)	−1^①^	0	0	−1^③^	−1^④^	CL
	WOMAC physical function score (6)	−1^①^	−1^②^	0	−1^③^	−1^④^	CL
	BPI-S score (5)	−1^①^	0	0	0	−1^④^	L
	30% pain reduction rate (4)	−1^①^	0	0	0	−1^④^	L
	50% pain reduction rate (4)	−1^①^	−1^②^	0	0	−1^④^	CL
	Weekly 24-h average pain score (3)	−1^①^	0	0	0	−1^④^	L
	Treatment emergent adverse events (5)	−1^①^	0	0	−1^③^	−1^④^	CL
	Serious adverse events (5)	−1^①^	0	0	−1^③^	−1^④^	CL
	Treatment discontinuation rate (5)	−1^①^	0	0	−1^③^	−1^④^	CL
Gao (2019)	WOMAC total score (5)	−1^①^	−1^②^	0	−1^③^	−1^④^	CL
	WOMAC pain score (4)	−1^①^	−2^②^	0	−1^③^	−1^④^	CL
	WOMAC stiffness score (4)	−1^①^	−2^②^	0	−1^③^	−1^④^	CL
	WOMAC physical function score (4)	−1^①^	−2^②^	0	−1^③^	−1^④^	CL
	PGI-I scores (5)	−1^①^	−1^②^	0	0	−1^④^	CL
	BPI-S score (5)	−1^①^	−1^②^	0	0	−1^④^	CL
	30% pain reduction rate (5)	−1^①^	0	0	0	−1^④^	L
	50% pain reduction rate (4)	−1^①^	−2^②^	0	0	−1^④^	CL
	Treatment emergent adverse events (5)	−1^①^	0	0	0	−1^④^	L
	Serious adverse events (5)	−1^①^	0	0	−1^③^	−1^④^	CL
	Treatment discontinuation rate (3)	−1^①^	−1^②^	0	−1^③^	−1^④^	CL
Osani (2019)	WOMAC pain score (5)	−1^①^	−1^②^	0	0	0	L
	WOMAC physical function score (5)	−1^①^	−1^②^	0	0	0	L
	Treatment emergent adverse events (5)	−1^①^	−2^②^	0	0	0	CL
	Serious adverse events (5)	−1^①^	0	0	0	0	M
	Treatment discontinuation rate (5)	−1^①^	0	0	0	0	M
	Gastrointestinal adverse event (5)	−1^①^	−1^②^	0	0	0	L
	Quality of life improvement (3)	−1^①^	0	0	0	0	M
	Improvement of depressive symptoms (2)	−1^①^	0	0	−1^③^	0	L
Qu (2020)	WOMAC total score (2)	−1^①^	−1^②^	0	−1^③^	−1^④^	CL
	WOMAC pain score (3)	−1^①^	0	0	0	−1^④^	L
	WOMAC stiffness score (3)	−1^①^	−1^②^	0	−1^③^	−1^④^	CL
	WOMAC physical function score (3)	−1^①^	−2^②^	0	0	−1^④^	CL
	BPI-S score (5)	−1^①^	0	0	0	−1^④^	L
	Dry mouth (2)	−1^①^	−1^②^	0	0	−1^④^	CL
	Drowsiness (2)	−1^①^	0	0	0	−1^④^	L
	Nausea (2)	−1^①^	−1^②^	0	0	−1^④^	CL
Chen (2021)	30% pain reduction rate (5)	−1^①^	0	0	−1^③^	−1^④^	CL
	50% pain reduction rate (5)	−1^①^	0	0	0	−1^④^	L
	Pain reduction average rate (5)	−1^①^	0	0	−1^③^	−1^④^	CL
	WOMAC pain score (4)	−1^①^	−2^②^	0	0	−1^④^	CL
	WOMAC stiffness score (6)	−1^①^	−1^②^	0	−1^③^	−1^④^	CL
	WOMAC physical function score (6)	−1^①^	−1^②^	0	0	−1^④^	CL
	SF-36 physical functional subscale (2)	−1^①^	−2^②^	0	−1^③^	−1^④^	CL
	SF-36 bodily pain subscale (2)	−1^①^	−2^②^	0	0	−1^④^	CL
	SF-36 role physical subscale (3)	−1^①^	−2^②^	0	−1^③^	−1^④^	CL
	PGI-I scores (5)	−1^①^	−1^②^	0	0	−1^④^	CL
	CGI-S scores (4)	−1^①^	−2^②^	0	−1^③^	−1^④^	CL
	Treatment emergent adverse events (5)	−1^①^	−2^②^	0	−1^③^	−1^④^	CL
	Serious adverse events (5)	−1^①^	0	0	−1^③^	−1^④^	CL
	BPI-I score (3)	−1^①^	−1^②^	0	−1^③^	−1^④^	CL
	BPI-S score (5)	−1^①^	0	0	0	−1^④^	L

Abbreviations: CL, critically low; L, low; M: moderate; H, high; ①, The design of the experiment with a large bias in random, distributive hiding or blind; ②, The confidence interval overlaps less, the heterogeneity test *P* is Critically small, and the *I* ^*2*^ is larger; ③, Confidence interval is not narrow enough; ④, Fewer studies are included and there may be greater publication bias.

### 3.7 Observation Index and Efficacy Evaluation

We summarize the information contained in the SRs, as reported in Table 7.

**TABLE 7:** Results of included SRs.

Author	Comparisons	Outcomes (n)	Total patient number in Intervention group/total patient number in control group or total participants in both groups, study number
Wang	Duloxetine 60–120 mg QD vs. Placebo	BPI-S score (3)	MD −0.88, 95% CI −1.11; −0.65, *p* < 0.00001 (490/502, *n* = 3)
		30% pain reduction rate (3)	RR 1.49, 95% CI 1.31; 1.70, *p* < 0.00001 (488/501, *n* = 3)
		50% pain reduction rate (3)	RR 1.69, 95% CI 1.27; 2.25, *p* = 0.0004 (488/501, *n* = 3)
		PGI-I scores (3)	MD −0.47, 95% CI −0.63; −0.30, *p* < 0.00001 (481/495, *n* = 3)
		WOMAC physical function score (3)	MD −4.25, 95% CI −5.82; −2.68, *p* < 0.00001 (480/497, *n* = 3)
		Adverse events (3)	RR 2.15, 95% CI 1.48; 3.11, *p* < 0.00001 (503/508, *n* = 3)
		Serious adverse events (3)	RR 1.30, 95% CI 0.48; 3.47, *p* = 0.61 (503/508, *n* = 3)
		Treatment emergent adverse events (3)	RR 1.32, 95% CI 1.16; 1.49, *p* < 0.00001 (503/508, *n* = 3)
		Treatment discontinuation rate (3)	RR 1.43, 95% CI 1.14; 1.78, *p* = 0.002 (503/508, *n* = 3)
Chen	Duloxetine 60–120 mg QD vs. Placebo	BPI-S score (5)	WMD −0.74, 95% CI −0.92; −0.57, *p* < 0.00001 (842/853, *n* = 5)
		Weekly 24-h average pain score (3)	WMD −0.76, 95% CI −0.96; −0.56, *p* < 0.00001 (564/559, *n* = 3)
		30% pain reduction rate (4)	RR 1.43, 95% CI 1.29; 1.59, *p* < 0.00001 (672/678, *n* = 4)
		50% pain reduction rate (4)	RR 1.71, 95% CI 1.46; 1.99, *p* < 0.00001 (672/678, *n* = 4)
		WOMAC stiffness score (6)	WMD −0.47, 95% CI −0.60; −0.34, *p* < 0.00001 (993/1003, *n* = 6)
		WOMAC physical function score (6)	WMD −4.44, 95% CI −5.24; −3.64, *p* < 0.00001 (995/1001, *n* = 6)
		Treatment emergent adverse events (5)	RR 1.31, 95% CI 1.20; 1.44, *p* < 0.00001 (880/882, *n* = 5)
		Serious adverse events (5)	RR 0.92, 95% CI 0.40; 2.11, *p* = 0.84 (880/882, *n* = 5)
		Treatment discontinuation rate (5)	RR 2.26, 95% CI 1.63; 3.12, *p* < 0.00001 (880/882, *n* = 5)
Gao	Duloxetine 60–120 mg QD vs. Placebo	BPI-S score (5)	MD −0.77, 95% CI −0.95; −0.59, *p* < 0.00001 (842/853, *n* = 5)
		30% pain reduction rate (5)	RR 1.42, 95% CI 1.30; 1.56, *p* < 0.00001 (844/855, *n* = 5)
		50% pain reduction rate (4)	RR 1.62, 95% CI 1.30; 2.02, *p* < 0.0001 (716/727, *n* = 4)
		PGI-I scores (5)	MD −0.48, 95% CI −0.59; −0.37, *p* < 0.00001 (835/849, *n* = 5)
		WOMAC total score (5)	MD −5.43, 95% CI −6.87; −3.99, *p* < 0.00001 (740/739, *n* = 5)
		WOMAC pain score (4)	MD −1.63, 95% CI −2.63; −0.63, *p* = 0.001 (726/731, *n* = 4)
		WOMAC stiffness score (4)	MD −0.58, 95% CI −0.75; −0.41, *p* < 0.00001 (726/732, *n* = 4)
		WOMAC physical function score (4)	MD −4.22, 95% CI −6.17; −2.28, *p* < 0.0001 (740/739, *n* = 4)
		Treatment emergent adverse events (5)	RR 1.32, 95% CI 1.20; 1.44, *p* < 0.0001 (879/882, *n* = 5)
		Serious adverse events (5)	RR 0.84, 95% CI 0.37; 1.90, *p* = 0.68 (879/882, *n* = 5)
		Treatment discontinuation rate (3)	RR 1.88, 95% CI 1.29; 2.75, *p* = 0.001 (487/494, *n* = 3)
Osani	Duloxetine 60–120 mg QD vs. Placebo	WOMAC pain score (5)	SMD –0.38, 95% CI –0.48; –0.28, P: no report
		WOMAC physical function score (5)	SMD –0.35, 95% CI –0.46; –0.24, P: no report
		Treatment emergent adverse events (5)	RR 1.53, 95% CI 1.21; 1.92, P: no report
		Serious adverse events (5)	RR 1.03, 95% CI 0.42; 2.54, P: no report
		Treatment discontinuation rate (5)	RR 2.17, 95% CI 1.57; 3.01, P: no report
		Gastrointestinal adverse event (5)	RR 4.43, 95% CI 3.45; 5.69, P: no report
		Quality of life improvement (3)	SMD 0.40, 95% CI 0.26; 0.53, P: no report
		Improvement of depressive symptoms (2)	SMD –0.09, 95% CI –0.26; 0.07, P: no report
Qu	Duloxetine 60–120 mg QD vs. Placebo	WOMAC total score (2)	MD −0.34, 95% CI −0.48; −0.20, *p* < 0.05 (392/388, *n* = 2)
		WOMAC pain score (3)	MD −0.41, 95% CI −0.54;−0.29, *p* < 0.05 (519/524, *n* = 3)
		WOMAC stiffness score (3)	MD −0.24, 95% CI −0.37;−0.12, *p* < 0.05 (519/524, *n* = 3)
		WOMAC physical function score (3)	MD −0.43, 95% CI −0.55;−0.31, *p* < 0.05 (536/532, *n* = 3)
		BPI-S score (5)	MD −0.38, 95% CI −0.48;−0.28, *p* < 0.05 (842/853, *n* = 5)
		Dry mouth (2)	RR 3.55, 95% CI 2.00; 6.29, *p* < 0.05 (382/378, *n* = 2)
		Drowsiness (2)	RR 3.23, 95% CI 1.88; 5.54, *p* < 0.05 (382/378, *n* = 2)
		Nausea (2)	RR 6.95, 95% CI 2.99; 16.15, *p* < 0.05 (382/378, *n* = 2)
Chen	Duloxetine 60–120 mg QD vs. Placebo	30% pain reduction rate (5)	MD −0.54, 95% CI −0.71; −0.37, *p* < 0.00001 (842/854, *n* = 5)
		50% pain reduction rate (5)	MD −0.87, 95% CI −1.07; −0.66, *p* < 0.00001 (842/854, *n* = 5)
		Pain reduction average rate (5)	MD −0.68, 95% CI −0.87; −0.48, *p* < 0.00001 (842/854, *n* = 5)
		WOMAC pain score (4)	MD −0.81, 95% CI −0.92; −0.69, *p* < 0.00001 (813/815, *n* = 4)
		WOMAC stiffness score (6)	MD −0.47, 95% CI −0.60; −0.34, *p* < 0.00001 (998/1004, *n* = 6)
		WOMAC physical function score (6)	MD −4.22, 95% CI −5.14; −3.30, *p* < 0.00001 (988/998, *n* = 6)
		SF-36 physical functional subscale (3)	MD 1.62, 95% CI 0.12; 3.13, *p* = 0.03 (409/417, *n* = 3)
		SF-36 bodily pain subscale (3)	MD 1.22, 95% CI 0.08; 2.35, *p* = 0.04 (409/417, *n* = 3)
		SF-36 role physical subscale (3)	MD 1.04, 95% CI −0.10; 2.18, *p* = 0.07 (409/417, *n* = 3)
		PGI-I score (5)	MD −0.48, 95% CI −0.58; −0.37, *p* < 0.00001 (867/874, *n* = 5)
		CGI-S score (4)	MD −0.34, 95% CI −0.44; −0.24, *p* < 0.00001 (717/731, *n* = 4)
		Treatment emergent adverse events (5)	RR 1.31, 95% CI, 1.20; 1.43, *p* < 0.00001 (880/882, *n* = 5)
		Serious adverse events (5)	RR 0.92, 95% CI, 0.40; 2.11, *p* = 0.84 (880/882, *n* = 5)
		BPI-I score (3)	MD −0.76, 95% CI,−0.96; −0.56, *p* < 0.00001 (453/471, *n* = 3)
		BPI-S score (5)	MD −0.74, 95% CI,−0.92; −0.57, *p* < 0.00001 (842/853, *n* = 5)

#### 3.7.1 Pain Reductions

Five SRs (Wang et al., 2015; Chen et al., 2019; Gao et al., 2019; Qu et al., 2020; Chen et al., 2021) reported BPI-S to describe that duloxetine could reduce pain in KOA Patients. All SRs showed that duloxetine was superior to the control group in reducing pain in KOA patients. The largest sample size (Chen et al., 2021) included 5 RCTs with a total of 1,695 patients (MD −0.74, 95% CI, −0.92; −0.57, *p* < 0.00001). The results were statistically and clinically significant.

#### 3.7.2 Improvements in Pain

Four SRs (Wang et al., 2015; Chen et al., 2019; Gao et al., 2019; Chen et al., 2021) reported 30% pain reduction rate and 50% pain reduction rate, which were denoted as moderate and substantial improvements separately. With nearly 30% pain reduction rate, the largest sample size (Chen et al., 2021) included 5 RCTs with a total of 1,696 patients (MD −0.54, 95% CI −0.71; −0.37, *p* < 0.00001). With about 50% pain reduction rate, the largest sample size (Chen et al., 2021) included 5 RCTs with a total of 1,696 patients (MD −0.87, 95% CI −1.07; −0.66, *p* < 0.00001). All SRs demonstrated that duloxetine was superior to the control group in the improvement of pain in KOA patients.

Four SRs (Gao et al., 2019; Osani and Bannuru, 2019; Qu et al., 2020; Chen et al., 2021) reported WOMAC pain score. The largest sample size included 4 RCTs with a total of 1,628 patients (MD −0.81, 95% CI −0.92; −0.69, *p* < 0.00001). 2 SRs (Gao et al., 2019; Qu et al., 2020) reported WOMAC total scores. 6 SRs reported WOMAC physical function score. The largest sample size (Chen et al., 2021) included 6 RCTs with a total of 1986 patients (MD −4.22, 95% CI −5.14; −3.30, *p* < 0.00001). 4 SRs (Chen et al., 2019; Gao et al., 2019; Qu et al., 2020; Chen et al., 2021) reported WOMAC stiffness score. The largest sample size (Chen et al., 2021) included 6 RCTs with a total of 2002 patients (MD −0.47, 95% CI −0.60; −0.34, *p* < 0.00001). These SRs’ consensuses were that the duloxetine group was more effective. The SR published in 2019 reported that duloxetine is efficient in managing chronic pain and loss of physical function but has no advantage in the treatment of joint stiffness. Meanwhile, statistically obvious differences in the variable between duloxetine and placebo were also demonstrated.

#### 3.7.3 Patient’s Global Impression

Three SRs (Wang et al., 2015; Gao et al., 2019; Chen et al., 2021) reported PGI-I. The largest sample size (Chen et al., 2021) included 5 RCTs with a total of 1741 patients (MD −0.48, 95% CI −0.58; −0.37, *p* < 0.00001). Besides, one SR (Chen et al., 2021) reported that the global impression of the patient measured by CCG-I was significantly improved with duloxetine (MD −0.34, 95% CI −0.44; −0.24, *p* < 0.00001).

#### 3.7.4 Quality of Life and Depressive Symptoms

One SR (Osani and Bannuru, 2019) reported duloxetine showed no significant effects on depression symptoms (SMD –0.09, 95% CI –0.26; 0.07, P: no report) whereas results on quality of life were statistically significant (SMD 0.40, 95% CI 0.26; 0.53, P: no report). In one SR (Chen et al., 2021), involving 3 RCTs and 826 patients, duloxetine was found to negatively influence the reduction of bodily pain (MD = 1.22; 95% CI 0.08; 2.35, *p* = 0.04) and physical functioning subscales (MD 1.62, 95% CI 0.12; 3.13, *p* = 0.03) of the SF-36. The SF-36 physical subscale also showed no indication of improvement (MD = 1.04, 95% CI −0.10; 2.18, *p* = 0.07).

#### 3.7.5 Safety

The main causes of TEAES in the duloxetine therapy group included constipation, nausea, sweating, cough, myalgia, joint pain, palpitations and dry mouth. The results of SRs revealed that duloxetine was associated with a high incidence of TEAES. The largest sample size (Chen et al., 2021) included 5 RCTs with a total of 1,762 patients (RR 1.31, 95% CI, 1.20; 1.43, *p* < 0.00001). Furthermore, all SRs agreed that there existed no obvious difference in the incidence of serious adverse events between the duloxetine and placebo groups. The largest sample size (Chen et al., 2021) included 5 RCTs with a total of 1,762 patients (RR 0.92, 95% CI, 0.40; 2.11, *p* = 0.84). 4 SRs (Wang et al., 2015; Chen et al., 2019; Gao et al., 2019; Osani and Bannuru, 2019) reported that duloxetine is significantly associated with treatment discontinuation rate. The largest sample size (Chen et al., 2019) included 5 RCTs with totally 1,762 patients (RR 2.26, 95% CI 1.63; 3.12, *p* < 0.00001).

## 4 Discussion

### 4.1 Summary of the Main Results

Admittedly, this is the first review of SRs exploring the efficacy and safety of duloxetine for the treatment of KOA. We critically evaluated the published SRs by adopting AMSTAR 2, ROBIS, PRISMA, and GRADE. In addition, the reporting quality according to the PRISMA checklist was relatively good, with a relatively complete manuscript structure and 4 SRs (Chen et al., 2019; Gao et al., 2019; Osani and Bannuru, 2019; Chen et al., 2021) being adequately reported by over 70%. However, in the grading results, the quality of evidence was poor, with all SRs assessed by AMSTAR 2 which had over one critical flaw. Thus, all SRs were rated very low. By adopting the ROBIS tool, the ratings for the two SRs (Chen et al., 2019; Gao et al., 2019) were unsatisfactory, suggesting that the conclusions based on the contained SRs may have difference from the true picture. GRADE results have revealed that duloxetine reduces pain in patients with KOA and improves joint function in those patients. Although all SRs appear to show the benefits of duloxetine, the results of the comprehensive review are not ideal. No definitive conclusions can be drawn. According to the published results, caution is required when recommending duloxetine as the treatment for patients with KOA.

### 4.2 Implications for Further Studies

The current overview introduces several challenges for producers of SRs that should be taken into consideration. The results of AMSTAR 2 tool and PRISMA checklist suggest that the methodological quality of SRs requires to be enhanced in the following areas. SRs should be registered in advance in the international preregistration database (PROSPERO) and should also detail the reasons for the type of study design, contributing to lower risk of bias in SRs. SRs should provide a comprehensive search strategy and focus on the search for grey literature and offer a detailed search strategy for at least one major database in order to the transparency of systematic evaluation. Literature exclusion lists and reasons should be provided to facilitate quality judgment and screening of selected literature. SRs should detail the fundamental features of the included studies, which is beneficial for understanding the comparability of study baselines. SRs should employ reasonable tools with the purpose of evaluating the inclusion risk of bias in the involved studies. Studies should completely describe issues such as funding information and conflicts of interest. In addition, researchers should conduct descriptions of other analytical methods like the sensitivity analysis and subgroup analysis as well as report evidence summaries in the GREAD summary of results form. While many items in AMSTAR 2 tool and PRISMA checklist are repeatable, the different purposes of each tool make them complementary, causing more comprehensive assessments. The ROBIS tool makes up for the lack by evaluating the risk of bias in SRs. Perry et al., 2021.

It could be discovered that the risk of bias was comparatively high in domains 2 and 4 of phase 2 when we adopted the ROBIS tool. In domain 2, we concentrated on identifying and selecting studies. In order to evaluate SR effectively, researchers must focus on whether they search a proper range of databases and electronic sources. As an alternative to searching databases, conference reports and clinical trial registration platforms need to be used to find relevant reports. In domain 4, there was a high risk of bias in the synthesis of findings. Although all data was synthesized, we were not capable of determining whether the necessary methods of data analysis and synthesis were followed before the SRs. As a result, some studies may not have been included in the synthesis. Moreover, it is essential to carry out a funnel plot or sensitivity analysis to assess the robustness of the findings as well as to minimize or address biases in primary studies in the synthesis.

As a result of the GRADE Tool, most indicators were rated as very low-quality evidence, implying variations in the findings. The main factors for downgrading included limitations and publication bias, followed by inconsistency. The downgraded limitations suggested that all studies were unclear or had large limitations in terms of randomization, allocation concealment, and blinding. In addition, future clinical trials should concentrate on a top-level design. It is most apparent from the low number of negative results and asymmetry of funnel plots that publication bias was present. The inconsistency was caused by the high heterogeneity of the included studies and the large I^2^ values after the merger, indicating that other analysis methods, like sensitivity analysis and subgroup analysis, should be performed to account for the heterogeneity.

### 4.3 Selection of Knee Osteoarthritis Outcome Indicators

The current study adopted the BPI scale for describing changes in pain scores. The BPI is primarily employed to assess pain in the past 24 h or the past 1 week. The main components of the assessment contain the level of pain, the type of pain, and the influence of pain on daily function. Based on our knowledge, the visual analogue scale (VAS) is the most commonly used scale to evaluate pain in KOA patients and features the highest reliability (Myles et al., 2017). In comparison with the VAS, the BPI measures pain intensity while testing the impact of pain on psychology, mood, and sleep, providing a more comprehensive assessment of pain (Poquet and Lin, 2016; Alizadeh-Khoei et al., 2017; Chiarotto et al., 2019). As a result, BPI is more suitable for use in KOA patients undergoing depression. The WOMAC, PGI-I, CGI-I, BPI, and SF-36 scales are comprehensive scales that each has its own focus. The WOMAC scale is categorized into three categories, respectively, pain, stiffness, and physical function. It is highly reliable and can effectively evaluate the course of disease and treatment effect in patients suffering from KOA. PGI-I is a global index that can be applied to assess a condition’s response to therapy (Viktrup et al., 2012; Bjelic-Radisic et al., 2018). PGI-I has only been tested on women undergoing stress incontinence. Apart from that, it has not been demonstrated that it applies to KOA patients. The Clinical Global Impressions scale is one of the most extensively applied scales in clinical trials in psychopharmacology (Mohebbi et al., 2018). The SF-36, a brief health questionnaire, provides a comprehensive overview of the respondents’ quality of life in eight areas (Brazier et al., 1992; Ware and Sherbourne, 1992). The result was reported by 1 SR, showing that duloxetine exerted a negative effect on improving the SF-36 physical function subscale and the physical pain subscale, without any statistically significant difference in the SF-36 role physical scale (Apolone and Mosconi, 1998; Lins and Carvalho, 2016). One SR (Osani and Bannuru, 2019) reported improvement in depressive symptoms, exhibiting no improvement in depressive symptoms in KOA patients with duloxetine. The result may be resulted from the small sample size and inaccurate conclusions due to the explicit exclusion of patients with depression in four studies and the exclusion of participants who were taking any other antidepressants in one study. In addition, we recommend adopting the Hamilton Rating Scale for Depression for describing the improvement in depressive symptoms in future studies (Hamilton, 1960; Zimmerman et al., 2013). Whether SF-36, PGI-I, and CGI-I can be used as indicators in order to assess the quality of life of KOA patients still needs to be further investigated. Inconsistent diagnostic criteria of SRs may generate inconsistent effectiveness evaluation criteria and ultimately influence the reliability of the results.

Despite some deficiencies in the 6 SRs, duloxetine may help to improve pain and depressive symptoms in KOA patients. Till present, numerous studies have shown that KOA patients with depressive symptoms, increased pain intensity, and functional limitations exhibit depressive symptoms in the context of musculoskeletal disorders. Duloxetine, a 5-hydroxytryptamine and norepinephrine reuptake inhibitor, may enhance the efficacy of depression and pain in KOA patients by providing pharmacological management of pain and depression as well as promoting bidirectional physical and psychological improvement. Besides, it is also recommended that future studies examine the effects of duloxetine in these populations with KOA and depression concomitantly. Moreover, large and well-controlled RCTs are still required with the purpose of assessing the long-term safety of duloxetine and its use as an alternative to conventional therapy.

### 4.4 Strengths and Limitations

First, this review is the first attempt to comprehensively review the methodology and quality of reporting of SRs on duloxetine for pain management in KOA patients with depressive symptoms. Secondly, we conducted the overview based on a predesigned protocol, lowering the probability of bias. However, there exist several limitations. There may have been studies in other languages missed because the study only employed computerized searches of English and Chinese publications. We only included SRs of RCTs. Moreover, some studies may generate negative results and not been published (Ioannidis, 2016; Heathers et al., 2019). Therefore, the number of included literatures was small, which may have generated the bias due to literature omission. In the evaluation using AMSTAR 2, PRISMA, ROBIS, and GRADE, although different researchers performed the evaluation and cross-checking, there may exist evaluation differences due to subjective differences in the scale entries. As a result, the results may not have been as accurate as they could have been since we were not capable of synthesizing all the evidence.

## 5 Conclusion

To conclude, duloxetine may become an effective therapy for improving pain and depressive symptoms in patients with KOA. However, this finding must be treated with caution given the generally low methodological and evidentiary quality of the involved researches. Future studies should concentrate on RCTs in patients undergoing concomitant OA and depression with the purpose of assessing the certain benefits of duloxetine in these populations. In addition, investigators need to improve the methodological quality, risk of bias as well as reporting quality of SRs to provide better quality evidence for evidence-based medicine.

## Data Availability

The original contributions presented in the study are included in the article/Supplementary Material, further inquiries can be directed to the corresponding author.
